# Identification of a potential homeodomain-like gene governing leaf size and venation architecture in birch

**DOI:** 10.3389/fpls.2024.1502569

**Published:** 2025-01-08

**Authors:** Xiuyan Bian, Chen Chen, Yang Wang, Chang Qu, Jing Jiang, Yao Sun, Guifeng Liu

**Affiliations:** ^1^ State Key Laboratory of Tree Genetics and Breeding, Northeast Forestry University, Harbin, China; ^2^ State Key Laboratory of Tree Genetics and Breeding, Chinese Academy of Forestry, Beijing, China; ^3^ Harbin Daowai District Bureau of Agriculture and Rural Affairs, Harbin, China; ^4^ Encyclopedia of China Publishing House, Beijing, China; ^5^ National Forestry and Grassland Administration Key State-owned Forest Areas Forest Resources Monitoring Center, Jiagedaqi, China

**Keywords:** homeodomain-like gene, leaf size, venation patterning, genetic regulatory network, co-expression network, birch

## Abstract

Leaf vein, an essential part of leaf architecture, plays significant roles in shaping the proper leaf size. To date, the molecular mechanisms governing leaf development including leaf venation patterning remains poorly understood in birch. Here, we performed the genome-wide identification of homeodomain-like (HD-like) superfamily genes using phylogenetic analysis and revealed the functional role of a potential HD-like gene in leaf growth and development using transgenic technology and transcriptomic sequencing. A total of 267 HD-like genes were identified based on *Arabidopsis* HD-containing transcription factors, which were members of KNOTTED1-like homeobox (KNOX) family, BELL1-like homeobox (BLH) family, Zinc finger-HD (ZHD) family, HD-leucine zipper (HD-Zip) family, Golden2, ARR-B, Psr1 (GARP) family, WUSCHEL-related homeobox (WOX) family, and Myeloblastosis (MYB) and MYB-like family. Further, 41 HD-like genes showing co-expression with marker genes related to leaf vascular tissues exhibited differential expression during primary vein development. Among them, a potential HD-like gene *(BpPHD4)* of GARP family served as a negative factor in governing leaf size and venation patterning. Compared to non-transgenic plants, *BpPHD4* repression transgenic plants showed increased leaf length, leaf width, leaf area, leaf thickness, spongy tissue thickness, stomata number, epidermal cell size, primary vein length, the distance between the secondary veins, and primary vein diameter, which was opposite to those of *BpPHD4* overexpression transgenic plants. Meanwhile, reduced expression levels of *BpPHD4* could remarkably promote phloem tissue development. Transcriptome analysis of *BpPHD4* overexpression transgenic plants showed two candidate genes (*Bpev01.c0518.g0018* and *Bpev01.c2797.g0002*) probably regulated by *BpPHD4.* To conclude, our findings contribute to a better understanding of HD-like superfamily genes and unravel the role of a potential HD-like gene in genetically controlling leaf size and venation patterning in birch, which provides clues to genetic improvement of woody plants with diverse geometric and topological properties of leaf vascular network.

## Highlights

- The study identified the homeodomain-like (HD-like) superfamily transcription factors and revealed a potential HD-like gene as a negative factor in regulating leaf size and venation patterns of birch plants.

## Introduction

Leaves of extant terrestrial plants are enormously diverse in their size and venation architecture, both of which play core roles in plant adaptation in ecosystems (Sack et al., 2012). The veins, which resemble the reticulated highway systems in leaves, are distributed and arranged in species-specific orders under natural environmental conditions (Aloni, 2021). The leaf vein systems are responsible for providing mechanical support for leaf growth, effectively transporting nutrients, and exchanging signaling information within the plant body (Zhu et al., 2020). The topological or geometric structures of leaf venation determine the growth and development of a leaf (Peng et al., 2022). Published studies have addressed the close association of leaf vascular arrangement to leaf size variations (Sack et al., 2012; Baird et al., 2021; Peng et al., 2022). Specifically, leaf venation properties predictively vary with leaf size during leaf development (Sack et al., 2012; Baird et al., 2021), finally leading to the benefits of prediction for leaf size (Peng et al., 2022). Although a large number of genetic factors responsible for initiating and developing leaf vascular tissues (Kang et al., 2007; Wenzel et al., 2007; Vanneste et al., 2011; Garrett et al., 2012; Donner and Scarpella, 2013; Sawchuk and Scarpella, 2013; Bar and Ori, 2014; Ichihashi et al., 2014; Naramoto et al., 2014; Thirulogachandar et al., 2017; Du et al., 2018; Yasui et al., 2018; Nikolov et al., 2019; Wu et al., 2021; Bian et al., 2024) or controlling leaf size (Gonzalez et al., 2012; Vercruysse et al., 2020; Karamat et al., 2021) have been well discovered, the developmental and molecular mechanisms underlying leaf venation architecture in relation to leaf size in woody plants, particularly birch trees, remain unclear.

Birch trees (*Betula* spp.), widely distributed across Europe and parts of Asia, are adapted to thrive in a wide range of habitats from boreal forests to temperate woodlands (Jerzy et al., 2020). The ecological and economic values of birch trees (Valobra and James, 1990; Miller et al., 1991; Gang et al., 2019), coupled with the roles in scientific research, underscore the need for thorough and systematic studies into their growth and developmental properties. The ordered and closely aligned patterns of leaf vascular tissues serve as a core determinant of many aspects of plant performance, which has key implications for forestry and conservation (Sack and Scoffoni, 2013; Bian et al., 2024). Our previous observations on the leaf veins reveal the variations that occurred between birch species (Qu et al., 2017, 2020). Compared to the lobed leaves, the diameter of primary vein from the unlobed leaves at same developmental stage is significantly decreased (Qu et al., 2017, 2020), suggesting the key roles of leaf veins in regulating birch leaf growth and development. As a result, understanding how leaf vein patterns develop in morphological and molecular details is of great importance in breeding new birch varieties with altered elements of leaf phenotypic traits. Combining with anatomical observation results, we have performed a weighted gene co-expression network constructed by the transcriptome data generated from birch primary veins at different developmental stages from immature to mature to identify candidate genes with potential roles in patterning leaf vein (Bian et al., 2024). With the aim of uncovering the functional roles of these identified genes in developing leaf morphology traits of birch trees, it is therefore necessary for us to carry out the present research at the molecular level.

Homeodomain-like (HD-like) superfamily proteins are a large group of essential TFs sharing a DNA binding domain in eukaryotes (Mannervik, 1999; Moreland et al., 2009; Kock et al., 2024), which are shown to control the expression of downstream genes to regulate diverse aspects of developmental and physiological processes (Bürglin, 2011; Tsuda and Hake, 2016; Chang and Lai, 2018). For instance, KNOTTED1-like homeobox (KNOX) and WUSCHEL-related homeobox (WOX) family genes are required for meristem maintenance and proper patterning of organ initiation (Williams, 1998; Hay and Tsiantis, 2010; Cao et al., 2015; Xiao et al., 2018; Tvorogova et al., 2021). In particular, ectopic expression of *OsWOX9A* transgenic plants exhibits narrow adaxially rolled rice leaves with enlarged bulliform cells and reduced large veins when compared to wild-type plants (Li et al., 2024). BELL1-like homeobox (BELL or BLH) family genes are expressed in the shoot apical meristem and form heterodimers to maintain its indeterminacy (Tsuda and Hake, 2016). HD-leucine zipper (HD-Zip) family genes play roles in developing trichome and non-root hair cells (Guan et al., 2008), controlling cotton fiber elongation (Shan et al., 2014), mediating panicle development (Huang et al., 2014a), establishing adaxial–abaxial polarity (Merelo et al., 2017), and patterning leaf venation (Moreno et al., 2018). Moreover, HD-containing genes from other families are responsible for flowering regulation (Soppe et al., 2000), leaf vein arrangement (Sun et al., 2002; Hong et al., 2011), vernalization mechanisms (Zografos and Sung, 2012), secondary cell wall formation (Hirano et al., 2013), wood formation (Petzold et al., 2018), and fatty acid biosynthesis (Jo et al., 2021). We therefore attempted to reveal the biological roles of HD-like superfamily genes in plant growth and development of birch trees based on the functional validations of HD-like genes in previously published literatures described above.

As mentioned earlier, biologists have made great efforts to uncover the genetic mechanisms of a class of HD-containing TFs in some plants. However, the genome-wide identification of HD-like superfamily TFs in woody plants is not yet understood. To the best of our knowledge, this was a novel paper to identify HD-containing TFs in birch. In the present study, we first identified 267 HD-like superfamily genes in birch genome due to the presence of *Arabidopsis* HD-containing proteins. In combination with our previous co-expression network constructed by the phenotypic traits and transcriptome data of primary veins at different developmental stages in *Betula pendula* “Dalecarlica” (Bian et al., 2024), a set of 41 HD-like genes showing co-expressed patterns with marker genes related to leaf vascular tissues exhibited upregulation or downregulation during primary vein development. Among these, a potential HD-like gene *BpPHD4* (*Bpev01.c0082.g0014*) was identified as an inhibiting factor in governing leaf size and venation patterning. After analyzing transcriptome data of *BpPHD4* overexpression transgenic plants, two candidate genes (*Bpev01.c0518.g0018* and *Bpev01.c2797.g0002*) were likely to be affected by *BpPHD4*. Overall, our work provides some valuable information for understanding the molecular role of an HD-like superfamily gene and contributes to genetic improvement of birch trees with diversified elements of leaf vascular pattern.

## Materials and methods

### Identification, phylogenetic. and expression analysis of HD-like superfamily genes in birch

The genome files of *A. thaliana* (TAIR10) were downloaded from Phytozome v13 (Goodstein et al., 2012). The genome files of *B. pendula* (v1.2 scaffolds) (Salojärvi et al., 2017) were downloaded from the CoGe comparative genomics platform (https://genomevolution.org/CoGe/). Full-length HD-like transcription factors in *A. thaliana* downloaded from the InterProScan website (Paysan-Lafosse et al., 2023) were used as queries to perform TBLASTN v2.7.1 software analysis against the birch genome database with an e-value of 0.00001. The resulting sequences were then subjected to InterProScan v5.0 software (Jones et al., 2014) with default parameters and were further searched against *Arabidopsis* genome using the BLASTP v2.7.1 software to obtain the putative HD-like genes in birch.

The MAFFT v7.475 program (Katoh et al., 2002) under the default parameters was used to perform the sequence alignment of newly identified birch and *Arabidopsis* HD-like proteins and the sequence alignment of newly identified birch HD-like superfamily members, respectively. Maximum likelihood (ML) phylogenetic trees were constructed using the IQTREE v1.6.12 software (Minh et al., 2020) with the sequence alignment of birch and *Arabidopsis* HD-like proteins and the sequence alignment of birch HD-like proteins, respectively. The reliability of our phylogenetic trees was determined by the bootstrap value of 1000 replicates.

We acquired the expression values of HD-like genes from our previous study of transcriptome data on primary veins at different developmental stages in *B. pendula* “Dalecarlica” (Bian et al., 2024). Heatmaps of gene expression levels were carried out by the pheatmap v1.0.12 package (Xu et al., 2023).

### The construction of co-expression network for HD-like genes involved in leaf vein development

Co-expression network analysis using the Weighted Gene Co-Expression Network Analysis (WGCNA) package (Langfelder and Horvath, 2008) was performed as previously described (Bian et al., 2024). To identify potential HD-like genes involved in leaf vein development, we analyzed the connectivity profiles of HD-like genes in METurquoise module and marker genes related to leaf vascular tissues identified in our previous study (Bian et al., 2024). Weight values of our co-expression network were set beyond 0.5 and were visualized using VisANT v5.0 (Hu, 2014).

### Plant materials and growth conditions

Micro-propagated plants of *B. platyphylla* were grown on 6‰ agar-solidified woody plant medium (WPM) with 3.0% sucrose under the pH ranging from 5.6 to 6.0. The growth chamber conditions were set as follows: 25 °C, 16 h of light per day, and a light density of ~5000 lux. Each line of birch plants was individually expanded to 30 young seedlings in a cultivation room. After the seedlings rooted by tissue culture for 40 days, they were transplanted to a vessel containing 12 chambers. The size of each chamber was 10 × 10 × 10 cm^3^. The substrates in chambers comprised a mixture of peat soil, vermiculite, and perlite (volume ratio = 5: 3: 2). All plants were grown under standard greenhouse conditions in the birch seed orchard of Northeast Forestry University in Harbin city, Heilongjiang Province, China.

### Vector construction of *BpPHD4*

The full-length coding sequence (CDS) of *BpPHD4* was amplified from young birch leaves by polymerase chain reaction (PCR) assay. The primers were listed in **Supplementary Table S1**. Overexpression vector 35S::*BpPHD4* was constructed using the cloned CDS sequence of *BpPHD4* by enzyme cleavage and ligation method. The sequences were digested with *BamH*I–*Pst*I and then purified and cloned into the *BamH*I–*Pst*I-digested pCAMBIA1300 vector at 16°C overnight. Specific sequences of *BpPHD4* were respectively identified using the NCBI Conserved Domain Databasetool (Lu et al., 2020) and were searched against birch genome using the TBLASTN v2.7.1 software. RNAi vector 35S::*anti-BpPHD4* was constructed using specific sequences of *BpPHD4* by the enzyme cleavage and ligation method. The forward and reverse specific sequences were sequentially digested with *BamH*I–*Spe*I and *Kpn*I–*Sac*I. After purification, sequences were respectively cloned into the *BamH*I–*Spe*I and *Kpn*I–*Sac*I-digested pFGC5941 RNAi vectors at 16°C overnight. The resulting recombinant plasmids were transformed into competent cells of *Escherichia coli* and then were transferred to agar-solidified lysogeny broth (LB) medium with kanamycin (50 mg/L) to screen for positive recombinant 35S::*BpPHD4* and 35S::*anti-BpPHD4* clones. After verifying the accuracy of these constructs by DNA sequencing, positive recombinant plasmids were introduced into EHA105 cells of *Agrobacterium tumefaciens* according to the method described by Liu et al. (2018).

### Genetic transformation in birch

The 35S::*BpPHD4* and 35S::*anti-BpPHD4* constructs were transformed into mature birch zygotic embryos via an *A. tumefaciens*-mediated transformation procedure as previously described (Gang et al., 2019). The transformed zygotic embryos were incubated on WPM medium supplemented with 0.8 mg/L 6-benzyladenine (6-BA), 0.02 mg/L naphthalene acetic acid (NAA), and 0.5 mg/L gibberellic acid (GA_3_) under dark conditions at 25°C for 3 days. The zygotic embryos were then incubated on selection medium (WPM supplemented with 0.8 mg/L 6-BA, 0.02 mg/L NAA, 0.5 mg/L GA_3_, 50 mg/L hygromycin, and 200 mg/L cefotaxime) to obtain callus tissues, followed by generating adventitious buds and the whole transgenic plants. Shoots of transgenic plants were cut into small species, which were further placed on the selection medium. The resistant transgenic shoots were transferred to regeneration medium (WPM supplemented with 1.0 mg/L 6-BA, 100 mg/L hygromycin, and 200 mg/L cefotaxime) for 20 days. After that, 2-cm-high resistant transgenic shoots were transferred to rooting medium (WPM supplemented with 0.2 mg/L NAA).

### Validation of birch transformants

Total DNA was isolated from young leaves of non-transgenic (NT), and *BpPHD4* overexpression and repression transgenic plants using the DNAquick Plant System (TIANGEN). The obtained DNA was selected as the template for PCR amplification using the primers listed in **Supplementary Table S2**. The PCR cycling conditions were as follows: 94°C for 2 min; 30 cycles of 94°C for 30 s, 58°C for 30 s, and 72°C for 1 min; 72°C for 8 min; and 16°C for 60 min. The PCR products were analyzed by 2.0% (w/v) agarose gel electrophoresis.

Total RNA was extracted from young leaves of NT and *BpPHD4* transgenic plants using the CTAB method and cDNA was generated as published previously (Bian et al., 2019a, 2019b). Quantitative real-time PCR (RT-qPCR) was performed using the quantitative SYBR green PCR Master Mix (Toyobo, Osaka, Japan) by an ABI 7500 Real-Time PCR system (USA), following the methods as previously described by Bian et al. (2019a, 2019b). Specific primers are listed in **Supplementary Table S2**. Relative expression levels of the targeted genes were calculated by the formula: relative expression level = 2^−ΔΔCT^ (Schmittgen and Livak, 2008) due to the presence of the housekeeping genes *18S rRNA* (FJ228477.1) and *α-tubulin* (GU476453.1). Each sample was conducted with three replicates.

### Phenotypic observations and statistical analysis of *BpPHD4* transgenic plants

The seedling height of *BpPHD4* transgenic and NT plants was measured every 25 days during the growth season from May to October. There were 12 seedlings of each transgenic line measured using a tower ruler.

To analyze the leaf morphological traits, the fourth leaves close to the apical buds from *BpPHD4* transgenic and NT plants were selected. A total of 20 leaves for each line were scanned using an Image Scanner III (ODYSSEY cLx, LI-COR, USA) and leaf length, leaf width, and leaf area were measured using the ImageJ software. After that, we adopted the leaf clearing method (Keating, 2009) to remove leaf pigments, followed by staining with 1% safranine O and scanning using an Image Scanner III (ODYSSEY cLx, LI-COR, USA). The number of secondary veins, major secondary angle to primary vein, distance between the secondary veins, and primary vein length was analyzed using the ImageJ software.

The fourth leaves close to the apical buds of *BpPHD4* transgenic and NT plants were respectively used for observing morphological characters of leaf epidermal cells and stomata. We removed the epidermis from the leaf abaxial surface that was observed under a stereomicroscope (Lumar. v12, ZEISS, Germany). Pavement cells of leaf epidermis were measured. Leaf tissues of 0.3 × 0.3 cm^2^ were collected from the region between the second and third secondary veins near the leaf base of *BpPHD4* transgenic and NT plants, followed by treatment in a high-vacuum instrument. We observed the stomata morphology under an S-3400N scanning electron microscope (HITACHI, Japan). Digital images were created with a scale bar.

The fourth leaf close to the apical bud of *BpPHD4* transgenic and NT plants was conducted for anatomical observation. Leaf tissues of 0.3 × 0.3 cm^2^ were collected from the region near the leaf margin located between the second and third secondary veins near the leaf base, and primary vein tissues of 0.3 × 0.3 cm^2^ were collected from the intersection between the primary vein and the second secondary veins close to the leaf base. All sampled tissues were treated as previously described by Huang et al. (2014b). A total of 30 sections were investigated using a microscope (Nikon 80i, Melville, NY, USA). The thickness of upper epidermis (UE), lower epidermis (LE), palisade tissue (PT), and spongy tissue (ST) was measured using the ImageJ software. Moreover, we performed anatomical observation on primary vein tissues, including ground tissue (GT), mechanical tissue (MT), xylem (X) tissue, and phloem (Ph) tissue.

All phenotypic data were statistically analyzed by the Statistical Package for the Social Sciences (SPSS) v24.0 software. The statistical significance was determined using one-way analysis of variance (ANOVA) with Duncan’s test under *p* < 0.05. The data were presented as average values ± standard error (SE).

### Transcriptome sequencing and data analysis

Three lines of *BpPHD4* overexpressed transgenic plants and one line of NT plants were selected. The third leaves close to the apical buds were collected for total RNA extraction. Three sets of biological replicates were performed. A total of 12 samples were subjected to transcriptome sequencing. RNA quality detection and cDNA library construction were performed as described previously (Bian et al., 2019a, 2019b). Paired-end sequencing was carried out at BMK Biotechnology Corporation (Beijing, China).

Raw reads were edited by discarding adaptor sequences, empty reads, and reads with unknown or low-quality bases using the fastp v0.23.2 software (Chen et al., 2018). Clean reads were mapped to the reference of *B. pendula* subsp. *pendula* genome v.1.2 (Salojärvi et al., 2017) using the HISAT2 v2.2.1 software (Kim et al., 2019). Transcript assembly and quantitation were evaluated using the StringTie v2.1.3b software (Pertea et al., 2016), and FPKM (fragments per kilo-bases per million mapped reads) values were applied to calculate gene expression levels. We performed the differential expression analysis using the DESeq2 v1.30.1 package (Love et al., 2014) with a criterion of *q*-value of 0.05 and an expression value of log_2_|fold change| >1 to characterize differentially expressed genes (DEGs). Classical Venn diagrams were constructed with the EVenn online software (Chen et al., 2021).

Functional annotation of proteins in *B. pendula* was performed through homolog searches (Bian et al., 2024). The methods for constructing a non-redundant functional annotation database using Gene ontology (GO) enrichment analysis were described in our previous study (Bian et al., 2024). GO enrichment analysis was analyzed using the clusterProfiler v3.18.0 package (Yu et al., 2012). GEne Network Inference with Ensemble of trees (GENIE3) (Huynh-Thu et al., 2010) and Gene Regulation Network Inference Using Time Series (GRENITS) (Morrissey et al., 2010) packages were used to generate hierarchical gene regulatory networks (GRNs). The GRNs were visualized using the Cytoscape v.3.7.2 software (Shannon et al., 2003). Heatmaps of gene expression levels were drawn by the pheatmap v1.0.12 package (Xu et al., 2023).

To identify the candidate genes regulated by *BpPHD4*, we applied the WGCNA package (Langfelder and Horvath, 2008) to perform co-expression network analysis of *BpPHD4* overexpressed transgenic plants. The eigengene values were used to indicate the association of genes between each module and phenotypic traits. Modules with a correlation coefficient greater than or equal to 0.7 and *p* < 0.05 were selected from the module–trait associations. The co-expression networks were constructed for *BpPHD4*, which were visualized using VisANT v5.0 software (Hu, 2014).

## Results

### Identification and phylogenetic analysis of HD-like superfamily genes in birch

To identify HD-like superfamily genes in birch, TBLASTN search was performed using *Arabidopsis* HD-like proteins downloaded from the InterProScan website as a query. After aligning against *Arabidopsis* protein sequences using the BLASTP program, we deleted redundant genes and identified 267 HD-like putative genes in birch. Analysis of these genes using the InterProScan v5.0 software showed 96.63% HD-like genes with annotated description of the HD-like superfamily (IPR009057) (**Supplementary Table S3**). To determine the accuracy of the other 3.37% HD-like genes, these protein sequences and other birch and *Arabidopsis* HD-like proteins were analyzed through multiple sequence alignments (**Supplementary Table S4**). According to the consistency of sequence alignments, these proteins were retained as putative HD-like genes in birch. Therefore, a total of 267 HD-like superfamily genes were identified in birch.

**Figure 1:**
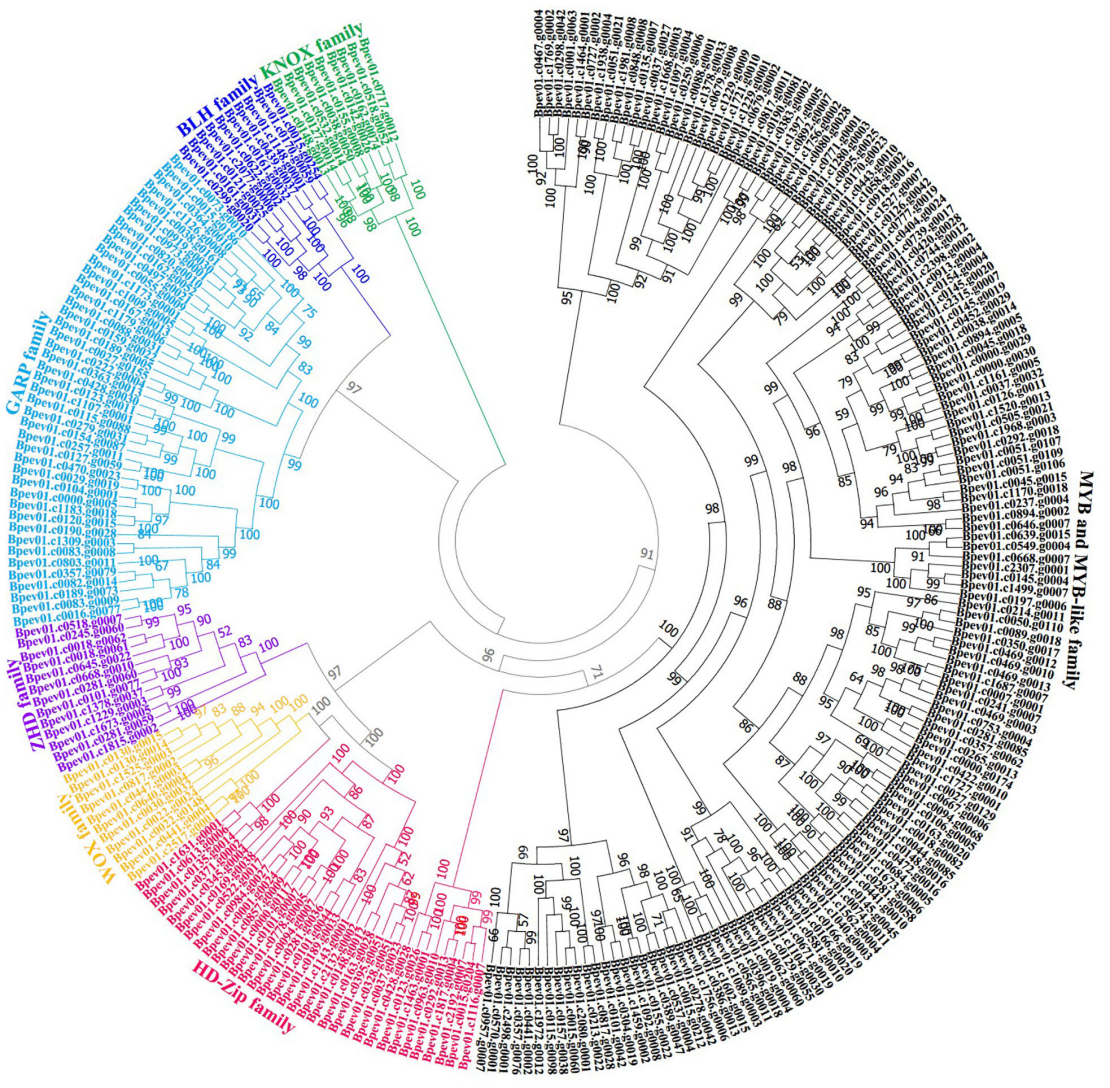
Phylogenetic trees of putative HD-like superfamily genes in birch. Genes from KNOX family are used as an outgroup. The reliability of this phylogenetic tree is evaluated by the bootstrap value of 1,000 replicates.

To understand the evolutionary relationship among birch HD-like proteins, we respectively constructed ML phylogenetic trees from sequences of 267 birch HD-like proteins and 271 *Arabidopsis* HD-like proteins (**Supplementary Figure S1**) and sequences of 267 birch HD-like proteins (**Figure 1**). The results showed that 267 birch HD-like proteins were divided into seven families (**Supplementary Table S5**). Of which, there were 9, 10, 11, 13, 33, 44, and 147 HD-like genes encoding KNOX family proteins, BLH family proteins, WOX family proteins, Zinc finger-HD (ZHD) family proteins, HD-Zip family proteins, Golden2, ARR-B, Psr1 (GARP) family proteins, and Myeloblastosis (MYB) and MYB-like family proteins, respectively. These results suggested the presence of HD-like genes in multiple gene families of birch trees.

### Prediction of a potential HD-like gene involved in leaf vein development

To predict genes relevant for regulating leaf vein development, we studied the profiles of these identified HD-like superfamily genes in our previous co-expression network, which was constructed by a METurquoise module of transcriptome sequencing on the primary veins at different developmental stages from immature to mature in *B. pendula* “Dalecarlica” (Bian et al., 2024). In our previous study, we respectively defined the first developmental stage, the second developmental stage, the third developmental stage, the fourth developmental stage, the fifth developmental stage, and the sixth developmental stage of primary veins as DS1, DS2, DS3, DS4, DS5, and DS6 (Bian et al., 2024). Based on the information of leaf vascular marker genes from our previously published study (Bian et al., 2024), a series of 41 genes showed co-expression patterns with 21 marker genes related to leaf vascular tissues when the threshold of weight values was set beyond 0.5 (**Supplementary Figure S2**). These marker genes respectively encoded the enzymatic products of ATRLCK VI_A3 (*Arabidopsis* receptor-like cytoplasmic kinase ATRLCK VI_A3), CML30 (Calmodulin-like 30), APL (Altered phloem development), PXL2 (Phloem intercalated with xylem-like 2), CDC2C (CDC2CAT), APX5 (Ascorbate peroxidase 5), GATA20 (GATA transcription factor 20), SMXL5 (SMAX1-like 5), ATPP2-A9 (Phloem protein 2-A9), AT4G10360 [TRAM, LAG1, and CLN8 (TLC) lipid-sensing domain containing protein], TMO6/DOF6 (Target of monopteros 6/DNA binding with one finger 6), HSP70-2 (Heat shock protein 70-2), GSTF4 (Glutathione S-transferase F4), LRD3 (Lateral root development 3), ATGUS1 (Glucuronidase 1), NEN1 (NAC45/86-dependent exonuclease-domain protein 1), ACI1 [ALCATRAZ (ALC)-interacting protein 1], VCC (Vasculature complexity and connectivity), TOL4 (TOM1-like 4), DA2 (DA (LARGE in Chinese) 2), and MTPB1 (Metal tolerance protein B1) (**Supplementary Figure S2**). Accordingly, we hypothesized the potential functions of these 41 HD-like genes in directing leaf vein development.

**Figure 2:**
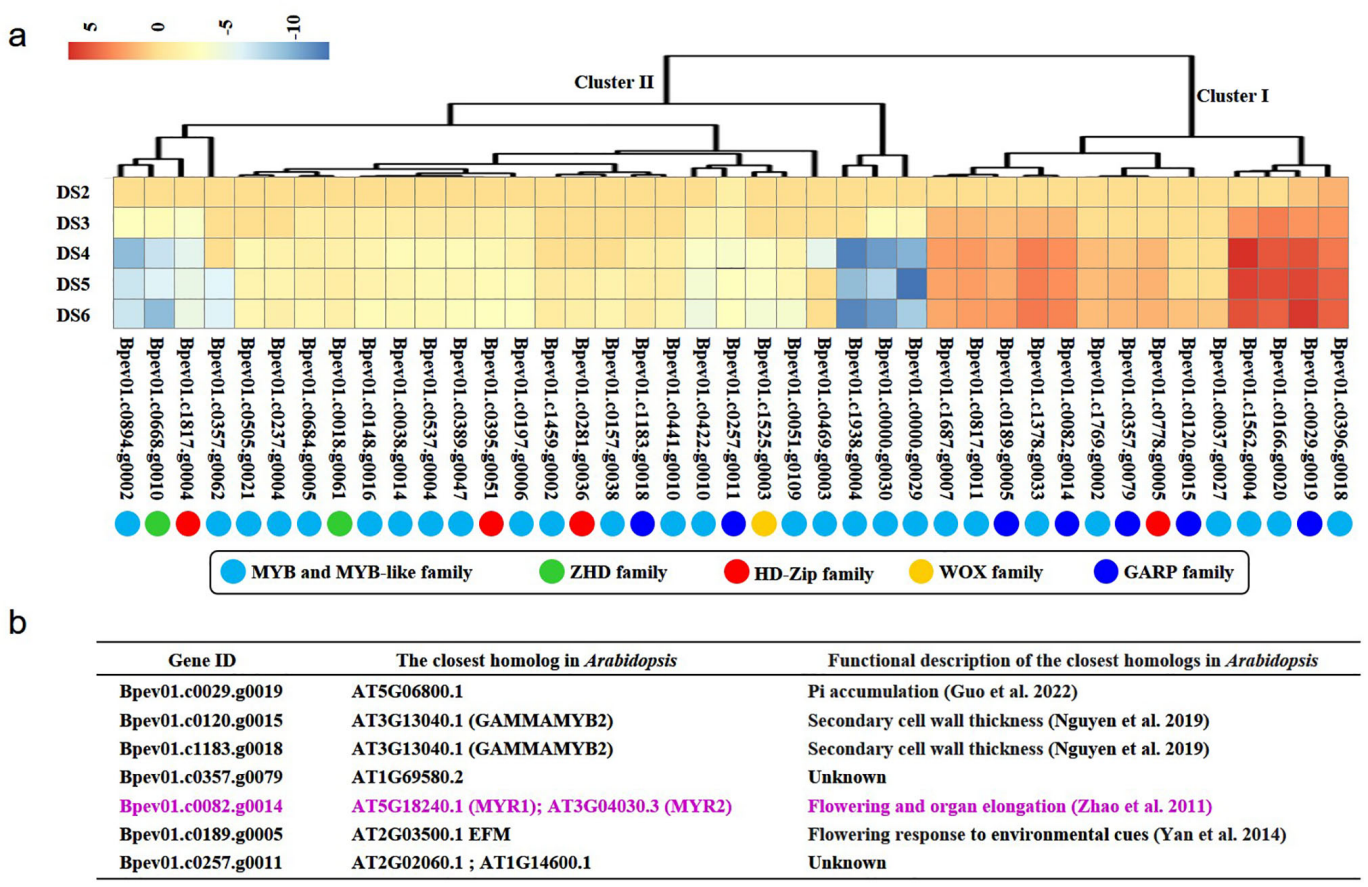
Identification of a candidate gene related to leaf vein development. **(A)** Expression patterns of HD-like genes during primary vein development. DS1, DS2, DS3, DS4, DS5, and DS6 respectively represent the first developmental stage, the second developmental stage, the third developmental stage, the fourth developmental stage, the fifth developmental stage, and the sixth developmental stage described in our previous study (Bian et al., 2024). Compared to DS1, the expression values of genes at DS2, DS3, DS4, DS5, and DS6 stage are calculated by the threshold of log_2_|fold change| > 1 and *q*-value of 0.05. MYB, myeloblastosis; ZHD, zinc finger-homeodomain; HD-Zip, homeodomain-leucine zipper; WOX, WUSCHEL-related homeobox; GARP, Golden2, ARR-B, Psr1. **(B)** Functional roles of the closest homologs of birch HD-like genes belonging to the GARP family.

Furthermore, expression levels of these 41 HD-like genes belonging to MYB, MYB-like, ZHD, HD-Zip, WOX, and GARP families were analyzed (**Figure 2A**). During the course of primary vein development, 34.15% HD-like genes from Cluster I were upregulated and 65.85% HD-like genes from Cluster II were downregulated (**Figure 2A**). Among all families, the proportion of upregulated genes belonging to the GARP family during primary vein development was the largest. Homolog searches (**Figure 2B**) showed that a potential HD-like gene *Bpev01.c0082.g0014*, a member of GARP family genes, was the closest homolog to MYR1 and MYR2 in *Arabidopsis*. *MYR1* and *MYR2* play important roles in *Arabidopsis* flowering and organ elongation and rice *myr1myr2* mutants exhibit abnormal leaf development and apical dominance (Zhao et al., 2011). In view of these lines of evidence, we thought this potential HD-like gene as a potential regulator in patterning organ formation and designated this gene as *BpPHD4* in this study.

### Generation of *BpPHD4* transgenic birch lines

To construct the overexpression and repression vectors of *BpPHD4*, we amplified the CDS and specific sequences. The recombinant 35S::*BpPHD4* and 35S::*anti-BpPHD4* constructs were verified by PCR amplification (**Supplementary Figure S3**), followed by DNA sequencing.

Mature birch zygotic embryos were infected with *Agrobacterium* EHA105 with targeted sequences of *BpPHD4* and were cultivated on selection medium with 50 mg/L of hygromycin and 300 mg/L of cephalosporin for 20 days to generate green callus from cut sites on zygotic embryos (**Supplementary Figures S4A, B**). After that, multiple transgenic shoots were generated from callus (**Supplementary Figure S4C**). Shoots 3 cm in length (**Supplementary Figure S4D**) were transferred to rooting medium (**Supplementary Figure S4E**) and were cultivated for 20 days (**Supplementary Figure S4F**). Three independent hygromycin-resistant transgenic plants of 35S::*BpPHD4* and 35S::*anti-BpPHD4* were respectively obtained.

PCR analysis was performed on individual hygromycin-resistant lines of 35S::*BpPHD4* and 35S::*anti-BpPHD4*, respectively. All resistant lines produced a 1,000-bp band with a *hygromycin-resistant* (*HygR*) gene, and the negative controls of double-distilled water and NT plants were detected without a 1,000-bp band (**Supplementary Figures S4G, H**). Hygromycin-resistant transgenic plants of 35S::*BpPHD4* and 35S::*anti-BpPHD4* were respectively designated as *BpPHD4* overexpression transgenic lines (H4 transgenic lines) and *BpPHD4* repression transgenic lines (RIH4 transgenic lines).

**Figure 3:**
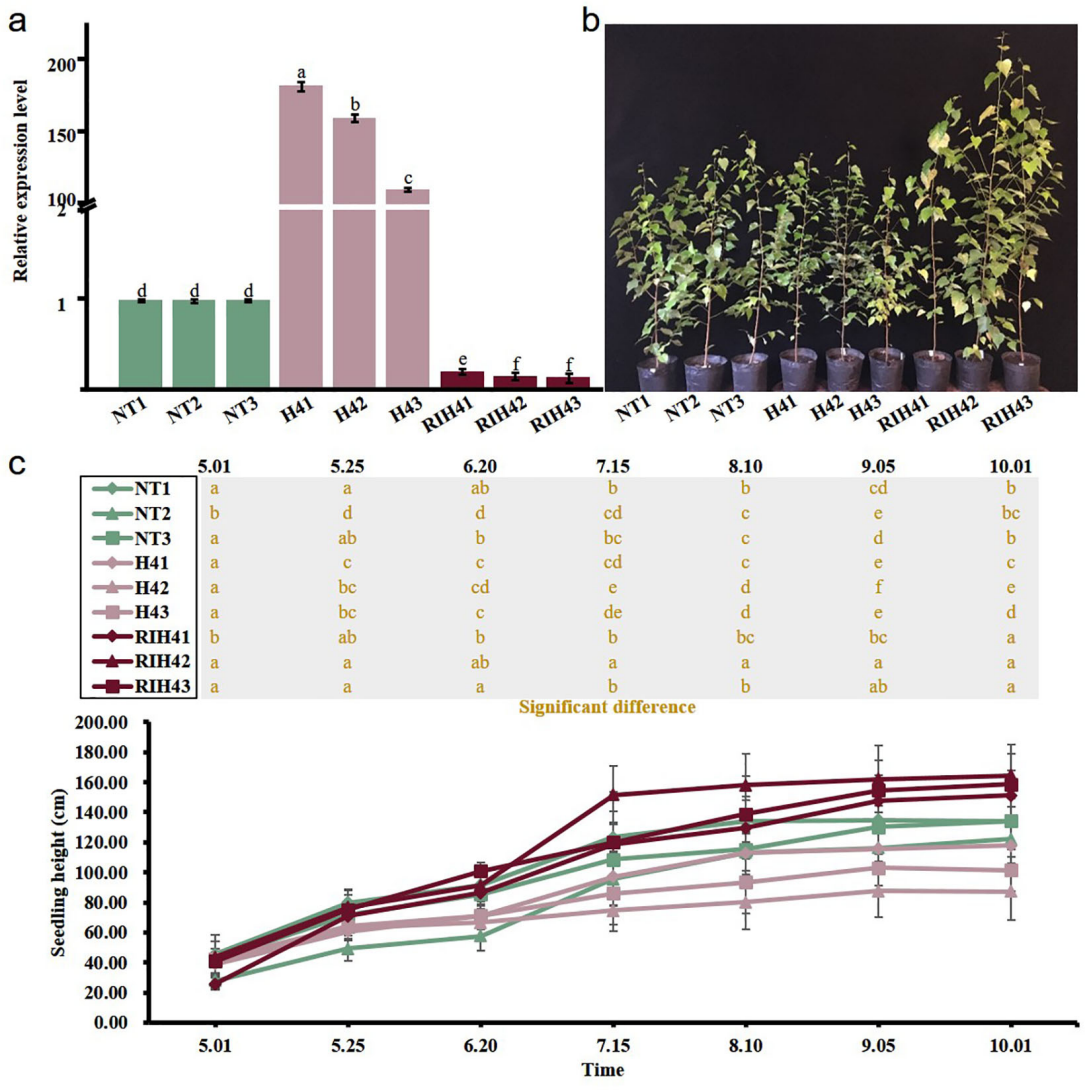
Characterization of *BpPHD4* transgenic birch plants and analysis of seedling height of *BpPHD4* transgenic birch seedlings. **(A)** Expression analysis of *BpPHD4* in H4 and RIH4 transgenic lines when compared to NT lines. **(B)** Image of 2-year-old NT and *BpPHD4* transgenic plants. **(C)** Seedling height of 2-year-old NT and *BpPHD4* transgenic plants during the growth season. The same letter indicates no significant difference, and different letters indicate a statistically significant difference when analyzed by one-way ANOVA and a multiple comparison using Duncan’s test at *p* < 0.05.

Analysis of RT-qPCR assay showed that three H4 transgenic lines had the increased expression levels of *BpPHD4* and three RIH4 transgenic lines had decreased expression levels of *BpPHD4* when compared to NT lines (**Figure 3A**). The expression of H4 transgenic lines was varied from 110.45- to 182.19-fold. The expression of RIH4 transgenic lines was varied from 0.13- to 0.20-fold. These findings therefore suggested that genetically modified plants of *BpPHD4* were successfully created. In the following study, three H4 transgenic lines were designated as H41, H42, and H43, respectively. Three RIH4 transgenic lines were designated as RIH41, RIH42, and RIH43, respectively.

### *BpPHD4* altered seedling height of birch plants

To evaluate the plant growth affected by *BpPHD4*, we measured the seedling height every 25 days during the growth season. Compared to NT lines, the seedling height of H4 and RIH4 transgenic lines gradually showed decreasing and increasing trends, respectively (**Figures 3B, C**). During the period of time, the relative seedling height of H4 and RIH4 transgenic lines was respectively 0.76 and 1.98 times as high as that of NT lines. These results suggested that *BpPHD4* was a factor repressing the plant growth of birch seedlings.

### *BpPHD4* affected the leaf development of birch plants

**Figure 4:**
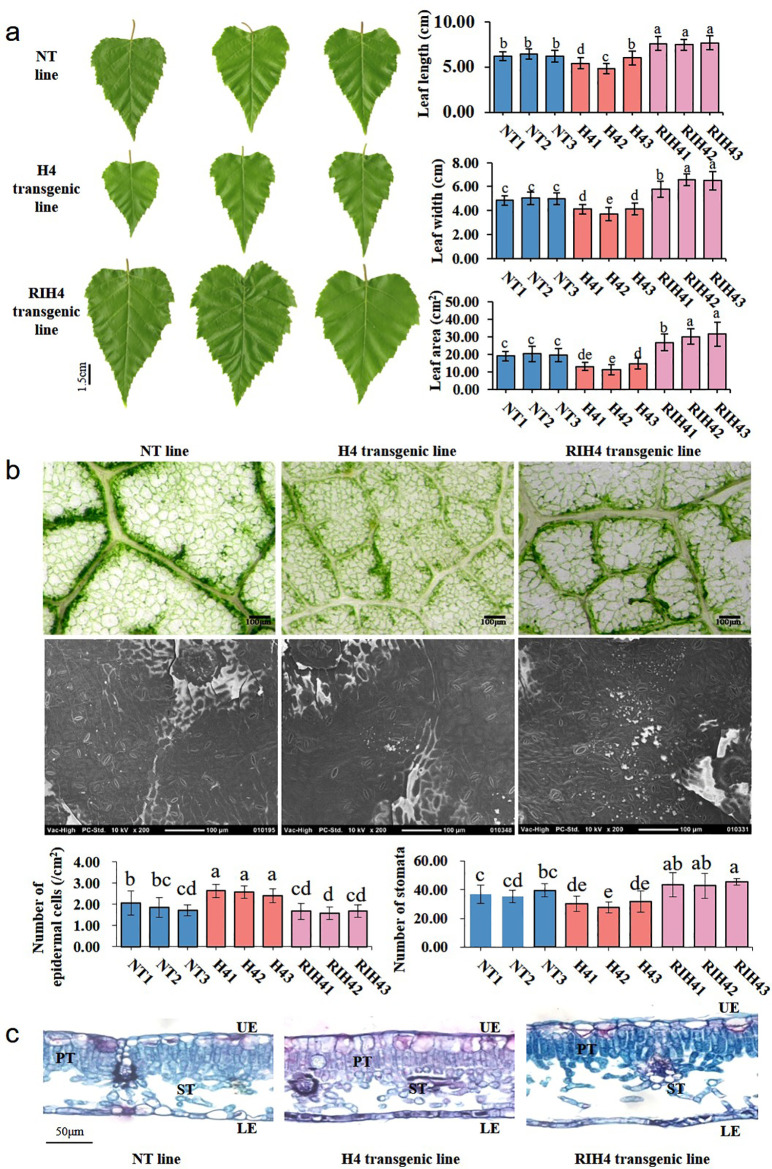
Observation on leaf morphology characters in birch. **(A)** Leaf length, leaf width, and leaf area of NT, H4, and RIH4 transgenic lines. **(B)** Observation on the epidermal cells and stomata and analysis of the number of epidermal cells and stoma in NT, H4, and RIH4 transgenic lines. **(C)** Anatomical observation on the leaves of NT, H4, and RIH4 transgenic lines. UE, upper epidermis; LE, lower epidermis; PT, palisade tissue; ST, spongy tissue. The same letter indicates no significant difference, and different letters indicate a statistically significant difference when analyzed by one-way ANOVA and a multiple comparison using Duncan’s test at *p* < 0.05.

Analysis of leaf morphological traits showed that leaf size was altered in *BpPHD4* transgenic plants (**Figure 4A**). Compared to NT lines, the average values of leaf length, leaf width, and leaf area of H4 transgenic lines were decreased by 13.49%, 19.71%, and 33.34%, respectively (*p* < 0.05). Compared to NT lines, the average values of leaf length, leaf width, and leaf area of RIH4 transgenic lines were increased by 20.69%, 26.72%, and 50.09%, respectively (*p* < 0.05).

Compared to NT lines, leaf epidermal cells of H4 and RIH4 transgenic lines, respectively, showed decreased and increased size (**Figure 4B**). The average number of leaf epidermal cells was significantly increased by 35.40% in H4 transgenic lines (*p* < 0.05) and was decreased by 12.67% in RIH4 transgenic lines (**Figure 4B**), as compared to NT lines. Transgenic lines of H4 and RIH4 appeared to have increased and decreased stomata size, respectively (**Figure 4B**). The average stomata number of H4 transgenic lines was 19.64% less than that of NT lines, while the average stomata number of RIH4 transgenic lines was 47.21% more than that of NT lines (**Figure 4B**).

Anatomical observation (**Figure 4C**) showed that the UE and LE of NT and *BpPHD4* transgenic lines were made up of one layer of closely associated cells. Cell size of UE and LE was irregular. As compared to LE, cell size of UE was increased. Contrary to H4 transgenic and NT lines, the PT of RIH4 transgenic lines exhibited loosely arranged cells. The ST of *BpPHD4* transgenic and NT lines was detected with intercellular space, of which RIH4 transgenic lines exhibited remarkably enlarged intercellular space of ST when compared to NT lines. Compared to NT lines, the average values of UE, LE, PT, ST, and leaf thickness in H4 transgenic lines were respectively decreased by 10.95%, 1.32%, 0.13%, 4.36%, and 4.09% (**Supplementary Table S6**). Compared to NT lines, the average values of UE, LE, PT, ST, and leaf thickness in RIH4 transgenic lines were respectively increased by 3.89%, 7.61%, 14.77%, 64.63%, and 34.79% (**Supplementary Table S6**), of which ST and leaf thickness reached significant difference (*p* < 0.05).

### *BpPHD4* related to leaf vein patterning in birch

**Figure 5:**
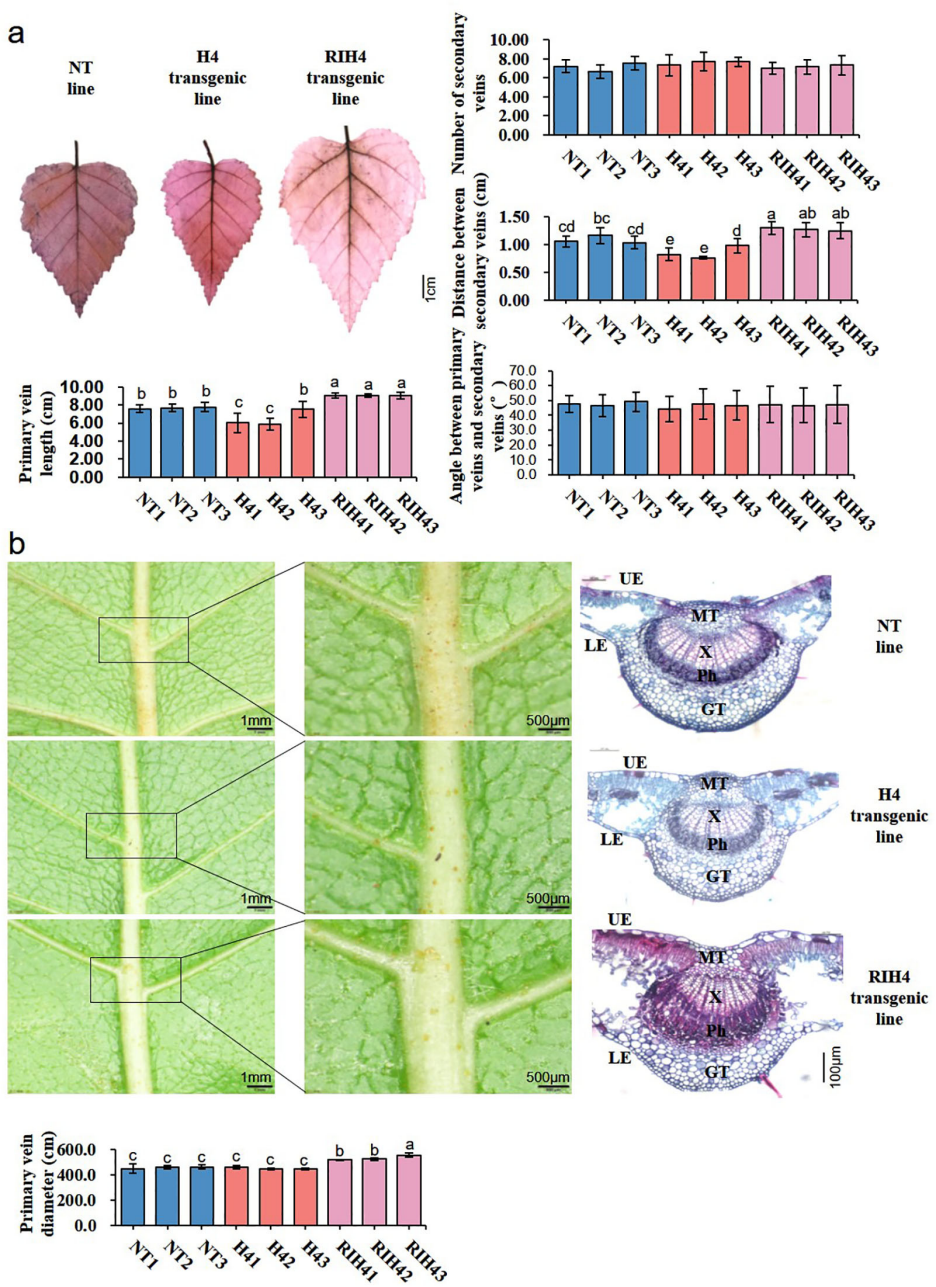
Observation on leaf vein patterns in birch. **(A)** Analysis of leaf vein traits in NT, H4, and RIH4 transgenic lines. The whole leaf stained by safranine O is used to investigate leaf vein systems. **(B)** Microscopic and anatomical observation on the primary vein of NT, H4, and RIH4 transgenic lines. The data are analyzed by the SPSS v24.0 software with Duncan’s test at *p* < 0.05 and presented as average values ± SE. The same letter indicates no significant difference, and different letters indicate a statistically significant difference. GT, ground tissue; Ph, phloem tissue; MT, mechanical tissue; X, xylem tissue; LE, lower epidermis; UE, upper epidermis.

Observation on leaf vein patterns showed that *BpPHD4* transgenic lines had altered leaf vein patterning (**Figure 5**). Compared to NT lines, primary vein length and distance between the secondary veins were significantly altered in *BpPHD4* transgenic lines (*p* < 0.05) (**Figure 5A**). Number of secondary veins and major secondary angle to primary vein showed no significant difference between *BpPHD4* transgenic and NT lines (*p* > 0.05) (**Figure 5A**), of which in comparison with NT lines, primary vein length was decreased by 15.70% in H4 transgenic lines and was increased by 17.69% in RIH4 transgenic lines, respectively. The distance between the secondary veins was 21.14% lower in H4 transgenic lines than that in NT lines, while it was 17.26% higher in RIH4 transgenic lines than that in NT lines.

Furthermore, we found anatomical traits of primary vein affected by *BpPHD4* (**Figure 5B**). Contrary to NT lines, RIH4 transgenic lines remarkably showed thickened phloem tissues of primary vein. Compared to NT lines, the primary vein diameter of H4 and RIH4 transgenic lines was respectively decreased and increased, which was consistent with scanning electron microscopic results (**Supplementary Figure S5**).

### Transcriptome analysis of *BpPHD4* overexpressed transgenic lines

To study the molecular role of *BpPHD4* in birch trees, the third leaves from 1-year-old NT and H4 transgenic lines were harvested. Analysis of transcriptome sequencing data (**Supplementary Table S7**) showed that raw reads ranged in size from 27,210,310 to 42,710,480. After quality checks, 20,847,226 to 30,869,378 clean reads and 3,097,081,491 to 4,581,159,902 clean bases were generated, with GC contents ranging from 44% to 47% and phred-like quality Q30 scores ranging from 96.10% to 98.12%. The average ratio of 87.52% clean reads could be successfully mapped to the reference genome. These results suggested the high quality of our tanscriptome data.

**Figure 6:**
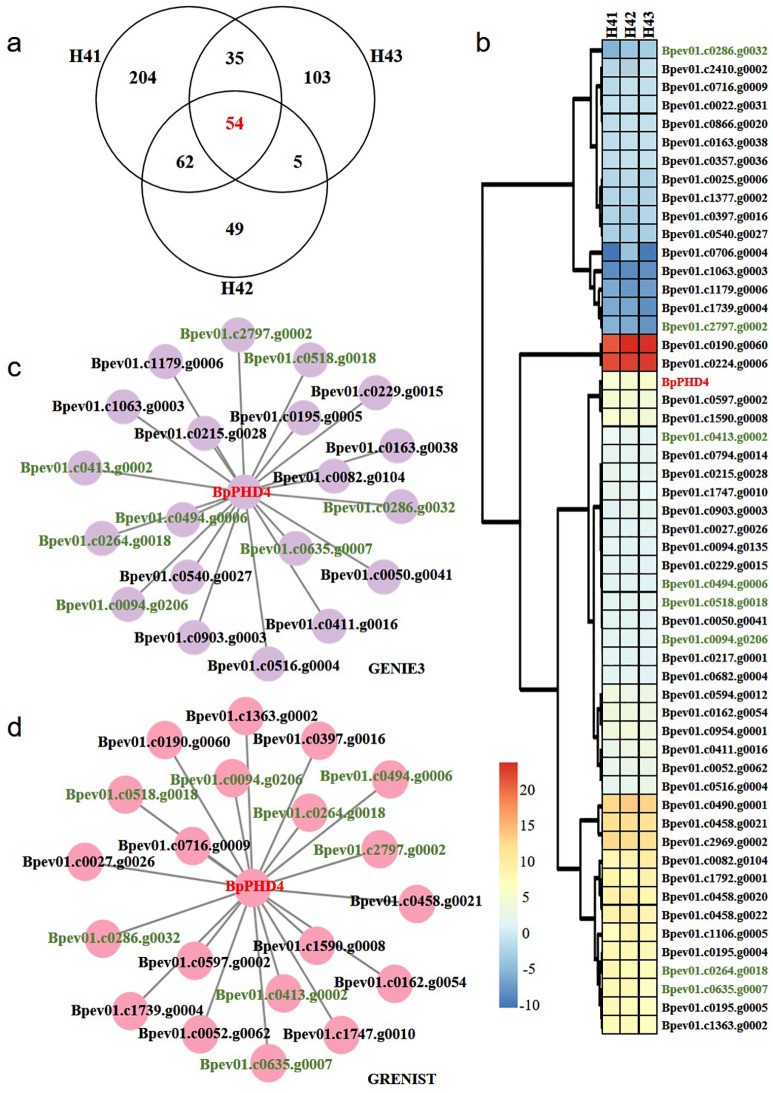
DEGs analysis and predication of candidate genes regulated by *BpPHD4*. **(A)** Venn diagram analysis of DEGs among three H4 transgenic lines. **(B)** Expression patterns of shared DEGs among three H4 transgenic lines. Expression values of genes are indicated by log_2_|fold change| values. **(C)** The GRN constructed by GENIE3 software. **(D)** The GRN constructed by the GRENITS software. Genes shown in green color are the intersection genes between two hierarchical networks.

Compared to NT lines, there were respectively 355 DEGs in the H41 transgenic line, 170 DEGs in the H42 transgenic line, and 197 DEGs in the H43 transgenic line. There were 54 shared DEGs among three H4 transgenic lines (**Figure 6A**). The expression patterns of these shared DEGs showed that 16 genes displayed decreased expression and 38 genes displayed increased expression (**Figure 6B**). Among these DEGs, *BpPHD4* was all upregulated in three H4 transgenic lines, confirming the reliability of our *BpPHD4* transgenic birch lines.

To investigate genes affected by *BpPHD4*, GRNs by shared DEGs were constructed using GENIE3 and GRENITS software (**Figures 6C, D**). Based on the probability or weight values, the top 20 genes were respectively chosen from two GRNs. Eight genes simultaneously occurred in two GRNs, namely, *Bpev01.c2797.g0002*, *Bpev01.c0094.g0206*, *Bpev01.c0635.g0007*, *Bpev01.c0286.g0032*, *Bpev01.c0518.g0018*, *Bpev01.c0413.g0002*, *Bpev01.c0494.g0006*, and *Bpev01.c0264.g0018*. These genes were mainly involved in calcium-dependent protein kinase (CDPK), early-responsive to dehydration stress (ERD) protein, leucine-rich repeat transmembrane protein kinase (LRRK), mitochondrial NADH dehydrogenase subunit 5 (ND5), plastid-encoded RNA polymerase-related development arrested protein, salicylate carboxy methyltransferase, caffeate O-methyltransferase (COMT), and protein of unknown function (DUF1005) (**Supplementary Table S8**).

### Co-expression network analysis of *BpPHD4* overexpressed transgenic lines

**Figure 7:**
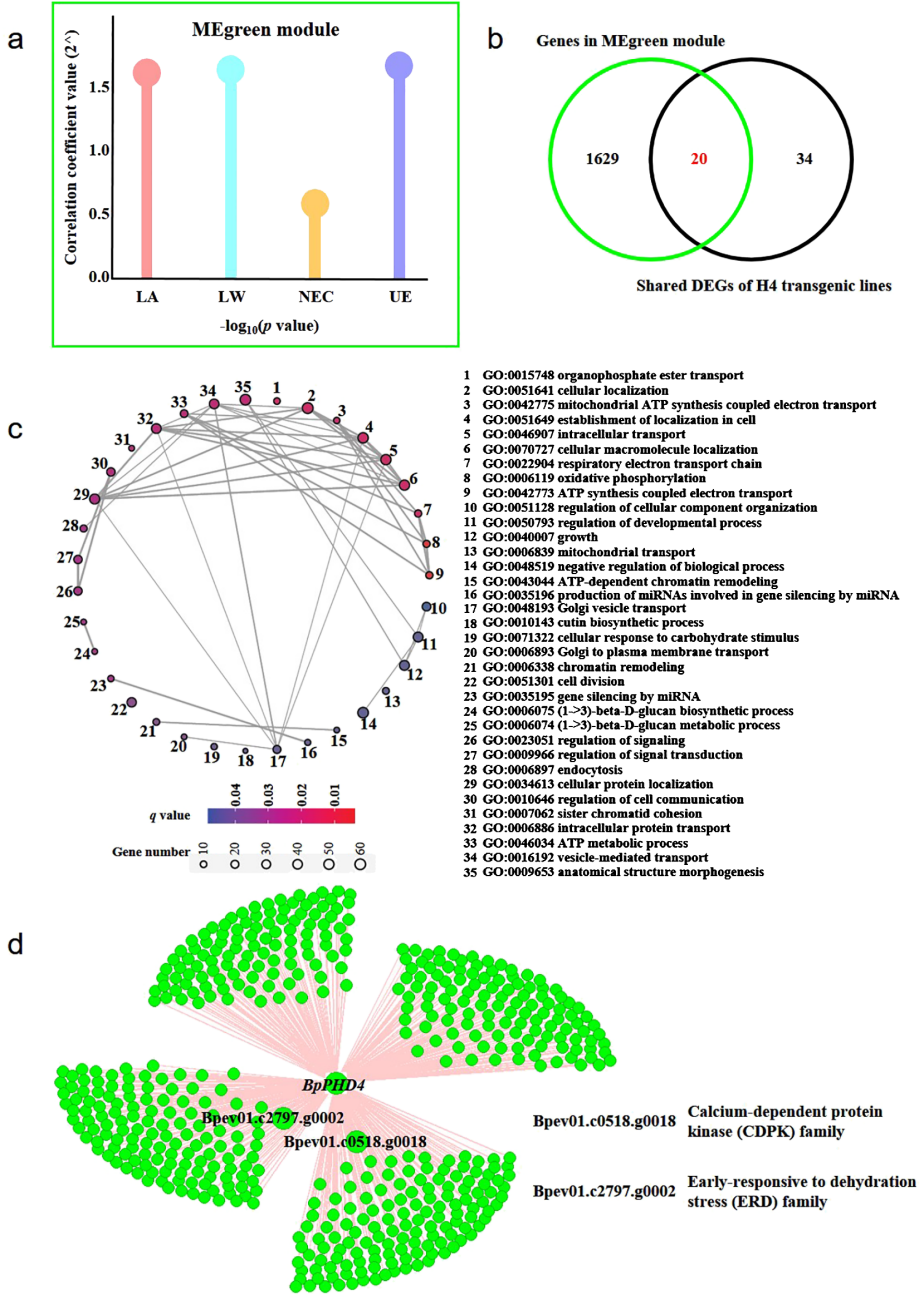
WGCNA of *BpPHD4* overexpressed transgenic lines. **(A)** Module traits of the MEgreen module. LW, leaf width; LA, leaf area; NEC, number of epidermal cells; UE, thickness of upper epidermis. **(B)** Venn diagram of genes in the MEgreen module and shared DEGs in H4 transgenic lines. **(C)** GO enrichment analysis of genes in the MEgreen module. **(D)** The co-expression network of two candidate genes and *BpPHD4* in the MEgreen module.

Based on transcriptome-based gene expression data and the phenotypic data of H4 transgenic lines, we used WGCNA software to perform co-expression network analysis (**Figure 7**; **Supplementary Figure S6**). A total of 42 modules related to phenotype data were identified. There was a MEgreen module simultaneously related to leaf area (LA), leaf width (LW), the number of epidermal cells (NEC), and the thickness of UE. The absolute value of correlation coefficient of the MEgreen module and phenotypes was beyond 0.7 (**Figure 7A**). Importantly, *BpPHD4* acted as a member of the MEgreen module. Venn diagram analysis showed that there were 20 genes simultaneously present between genes of the MEgreen module and shared DEGs of H4 transgenic lines (**Figure 7B**).

GO enrichment analysis (**Figure 7C**) showed that genes from the MEgreen module were mainly related to the negative regulation of biological process (GO:0048519), cellular protein localization (GO:0034613), vesicle-mediated transport (GO:0016192), anatomical structure morphogenesis (GO:0009653), cellular localization (GO:0051641), establishment of localization in cell (GO:0051649), intracellular transport (GO:0046907), cellular macromolecule localization (GO:0070727), and cell division (GO:0051301) (*q* < 0.05).

In the MEgreen module, we found two genes (*Bpev01.c0518.g0018* and *Bpev01.c2797.g0002*) from two GRNs constructed by shared DEGs that were co-expressed with *BpPHD4* (**Figure 7D**), of which *Bpev01.c0518.g0018* was a member of the CDPK gene family (**Supplementary Table S8**). *Bpev01.c2797.g0002* was a member of the ERD gene family (**Supplementary Table S8**). Co-expression analysis of the MEgreen module showed that these two genes were closely correlated with leaf area, leaf width, epidermal cell number, and UE thickness of *BpPHD4* overexpressed transgenic plants. These results suggested that these two genes were potentially regulated by *BpPHD4* in birch.

## Discussion

Leaf vein systems, showing great pattern diversification, are different types of vascular bundles distributed on the leaves in land plants (Hickey, 1973; Adams et al., 2018). This reticulated venation network takes the responsibility for transporting nutrients and water molecules, providing mechanical support for leaves and exchanging a wide range of signals within a living plant body, finally leading to the benefits of improving plant performance (Zhu et al., 2012; Sack and Scoffoni, 2013; Pagano et al., 2016). Leaf vascular tissues usually mirror the surrounding leaf size (Peng et al., 2022). A comprehensive understanding of the genetic regulation underlying leaf vein networks is endowed with key implications for genetic modification of woody species (Sack and Scoffoni, 2013). In the present study, on account of the information of *Arabidopsis* HD-containing proteins, we identified the members of HD-like superfamily in birch. Through analyzing our previous co-expression network closely associated with the phenotypic traits of the primary vein (Bian et al., 2024), a set of 41 HD-like genes was likely to exert roles in leaf developmental regulation. Among these TFs, a potential HD-like gene *BpPHD4* was characterized to encode a GARP family protein negatively mediating leaf size and venation patterning. Furthermore, we proposed the occurrence of two candidate genes directly or indirectly influenced by overexpression of *BpPHD4* in birch. Our study results could shed light on HD-like superfamily genes and contribute to genetic improvement of birch trees with altered geometric and topological characteristics of the leaf vein network.

It is common knowledge that TFs can bind to the promoter cis-regulatory elements of specific downstream target genes to activate or inhibit their expressions (Lagan and Gaëlle, 2008; Moon and Hake, 2011). HD-containing proteins are a type of TFs (Mannervik, 1999; Moreland et al., 2009; Kock et al., 2024). In our study, a total of 267 HD-like superfamily genes were identified in the birch genome, which mainly encoded KNOXs, BLHs, ZHDs, HD-Zips, GARPs, WOXs, and MYB and MYB-like proteins. These results suggested the HD-like genes present in multiple gene families of birch trees, consistent with prior studies (Hosoda et al., 2002; Guan et al., 2008; Hay and Tsiantis, 2010; Huang et al., 2014a; Tsuda and Hake, 2016; Zhang et al., 2020; Tvorogova et al., 2021). To the best of our knowledge, this was a novel article in birch identifying HD-like superfamily members at the genome-wide level.

With the aim of breeding new plant varieties, researchers have made great efforts to uncover genetic factors and hormonal molecules for initiating and arranging leaf vein networks (Kang et al., 2007; Scarpella et al., 2006; Wenzel et al., 2007; Garrett et al., 2012; Donner and Scarpella, 2013; Naramoto et al., 2014; Thirulogachandar et al., 2017; Yasui et al., 2018; Zhang et al., 2020; Linh and Scarpella, 2022; Duarte et al., 2023; Du et al., 2023; Bian et al., 2024; Chen et al., 2024; Scarpella, 2024). In particular, most of the HD-containing proteins play important roles in organ patterning and development (Guan et al., 2008). In this study, 41 HD-like genes encoding MYBs, MYB-like proteins, ZHDs, HD-Zips, WOXs, and GARPs were found to show co-expression patterns with leaf vascular marker genes (**Figure 2A**). According to the roles of these marker genes related to vascular tissues as described previously (Tsuda et al., 2017; Melnyk et al., 2018; Liu et al., 2022), these identified HD-like genes were thought to be potential regulators of leaf vascular development in birch. These results could be confirmed by the variations of leaf vein arrangement caused by genes possessing an HD-like domain in rice and *Arabidopsis* (Sun et al., 2002; Hong et al., 2011; Hirano et al., 2013; Moreno et al., 2018; Li et al., 2024).

Some previous studies show that TFs can affect the formation, differentiation, and connection of leaf veins by targeting the promoter sequences of specific downstream genes (Byrne et al., 2000; Barkoulas et al., 2007; Lagan and Gaëlle, 2008; Zhang et al., 2020), leading to the species-specific arrangement of leaf venation systems (Moon and Hake, 2011), of which HD-like genes play critical roles in governing a wide range of plant developmental and physiological processes (Bürglin, 2011; Tsuda and Hake, 2016; Chang and Lai, 2018). In our work, *BpPHD4* was identified as a member of HD-like superfamily genes (**Figure 1**; **Supplementary Figure S1**) and its transgenic plants displayed altered plant height and leaf phenotypic traits. *BpPHD4* showed the closest homology to *Arabidopsis* MYR1 and MYR2 in our phylogenetic tree responsible for repressing flowering and organ elongation partly through their regulatory effect on hormone levels (Zhao et al., 2011), further implying the roles of *BpPHD4* in genetically controlling birch growth and organ development.

Compared to NT lines, *BpPHD4* repression transgenic plants showed increased leaf length, leaf width, leaf area, leaf thickness, ST thickness, stomata number, and epidermal cell size. Along with leaf size, *BpPHD4* repression transgenic plants also had increased values of the primary vein length, the distance between the secondary veins, and the primary vein diameter. Overexpression of *BpPHD4* in birch led to the opposite phenotypes. These results indicated the negative effects of *BpPHD4* on shaping leaf development. One possible reason was that *BpPHD4* was a member of the co-expression network in a METurquoise module negatively correlated with phenotypic traits of the primary vein (Bian et al., 2024), suggesting the involvement of *BpPHD4* in inhibiting leaf venation patterning. The positive correlation between leaf vein traits and leaf size occurred in our *BpPHD4* transgenic plants, which was in agreement with the relation of leaf venation architecture to leaf size across leaf habits and vein types in subtropical woody plants (Peng et al., 2022). Many exogenous and endogenous factors have a great impact on leaf growth and development, the association of which to ST cells (McConnell et al., 2001; Husbands et al., 2009; Szakonyi et al., 2010), stomata number (Tuzet, 2011), and cell number (Schulz et al., 2014) has been previously documented. Considered together, the variations in leaf phenotypic properties could be attributed to the overexpression or repression levels of *BpPHD4* in birch.

Plant vascular tissues are composed of specialized cell types of xylem and phloem, which can be separated by the cambium (Wolf and Lohmann, 2019). The xylem and phloem cells typically located in a central region of the organ are of great significance in transporting water and nutrients (Lambers and Oliveira, 2019). Cambium tissue is home to dividing cells, which drives the expansion of the xylem to form wood and the phloem to form bast (Jouannet et al., 2015), thereby optimizing plant performance. Contrary to NT lines, increased phloem tissues remarkably occurred in *BpPHD4* repression transgenic lines, suggesting that *BpPHD4* played a key role in thickening phloem tissues in birch. As mentioned earlier, *BpPHD4* was the closest homologous to MYR1 and MYR2 in *Arabidopsis* specifically expressed in phloem tissues (Zhao and Beers, 2013), in favor of supporting the functional roles of *BpPHD4* in developing birch leaf vein possibly through phloem-thickening mechanisms. During an initial growth phase that precedes radial expansion, certain phloem cells at the periphery of the vascular tissue act as “organizers”—cells that promote the division of nearby cells (Miyashima et al., 2019), suggesting the roles of developing phloem cells in activating vascular development. Our results of phloem-thickening phenomena appeared to contribute to venation outgrowth in birch, which was similar to the theory of plant-thickness mechanism *Arabidopsis* roots (Wolf and Lohmann, 2019).

Leaves are enormously diverse in their size and venation architecture (Peng et al., 2022). Leaf size is an important determinant of the trade-off between carbon assimilation and water-use efficiency and is associated with energy exchange across different climates (Wright et al., 2017; Li et al., 2020). Leaf venation is the main structure for its function in support and transportation, thereby determining leaf outgrowth (Agustí and Blázquez, 2020; Samanta et al., 2021). Peng et al. (2022) propose the strong correlations of the major vein density and the ratio of major to minor vein density with leaf size across 39 woody species within a subtropical forest. We aimed to reveal he coordinated regulation of leaf vein development and leaf size strictly controlled by genetic effectors or their intrinsic regulatory networks. Based on the construction of gene regulatory and co-expression networks by our transcriptome data of *BpPHD4* overexpression transgenic plants, we found two candidate genes (*Bpev01.c0518.g0018* and *Bpev01.c2797.g0002*) probably regulated by *BpPHD4* in birch. Co-expression network analysis showed that these two genes were closely correlated with leaf area, leaf width, epidermal cell number, and UE thickness. These results enabled us to hypothesize the key role of these two genes in leaf size control, including leaf vascular development.

Sequence analysis showed that *Bpev01.c0518.g0018* and *Bpev01.c2797.g0002* belonged to the CDPK gene family and ERD gene family, respectively. In plants, CDPK family genes act as essential factors in the regulation of plant growth and development and abiotic and biotic stress tolerance (Asano et al., 2012; Xiao et al., 2016). For instance, *CDPK28* is characterized as a critical regulator in *Arabidopsis* stem elongation and vascular development (Matschi et al., 2013). In addition to *ERD* genes, they are a group of genetic factors in mediating leaf development (Rai et al., 2016; Wu et al., 2024). In *Brassica juncea*, *BjERD4* transgenic plants showed abnormal phenotypic traits in leaf number and leaf area (Rai et al., 2016). In *Arabidopsis*, phosphorylation of two amino acid sites on one stress-responsive and senescence-induced gene *ERD7* can promote age-dependent and stress-induced leaf senescence through disturbing the accumulation of hydrogen peroxide (Wu et al., 2024). Accordingly, we proposed that these two candidate genes appeared to be controlled by *BpPHD4*, playing an important role in affecting leaf development and venation patterning in birch.

## Conclusion

The design and function of leaf venation are endowed with critical characteristics for leaf performance, which is strictly controlled by common genetic factors. To gain insight into the molecular function of HD-like genes in leaf development, we identified a total of 267 HD-like superfamily genes and found that 41 HD-like genes showing co-expression with marker genes related to leaf vascular tissues exhibited differential expression patterns during primary vein development. Among them, a potential HD-like gene (*BpPHD4*) was identified as a negative factor in governing leaf size and venation patterning, the repression of which remarkably thickens the phloem tissue of the primary vein. Based on the construction of GRNs and co-expression networks by our transcriptome data of *BpPHD4* overexpression transgenic plants, two candidate genes (*Bpev01.c0518.g0018* and *Bpev01.c2797.g0002*) were probably regulated by *BpPHD4* in birch. These findings provide a comprehensive understanding of leaf size regulation and serve as references for genetic modification of leaves with diverse leaf vascular systems in woody plants.

## Data Availability

The datasets presented in this study can be found in online repositories. The names of the repository/repositories and accession number(s) can be found in the article/**Supplementary Material**.

## Supplementary material

Supplementary Figure 1 Phylogenetic tree of HD-like superfamily genes in birch and *Arabidopsis*. Genes in KNOX family are used as an outgroup. The reliability of this phylogenetic tree is evaluated by the bootstrap value of 1000 replicates. Black solid triangle denotes the HD-like gene in *Arabidopsis*. Black solid circle denotes the HD-like gene in birch.

Supplementary Figure 2 Co-expression network of HD-like genes and marker genes related to leaf vascular tissue. Pink rectangle represents the HD-like genes, orange circle the marker genes related to leaf vascular tissues, ATRLCK VI_A3 *Arabidopsis* receptor-like cytoplasmic kinase ATRLCK VI_A3, CML30 calmodulin-like 30, APL altered phloem development, PXL2 phloem intercalated with xylem-like 2, CDC2C CDC2CAT, APX5 ascorbate peroxidase 5, GATA20 GATA transcription factor 20, SMXL5 SMAX1-like 5, ATPP2-A9 phloem protein 2-A9, AT4G10360 TRAM, LAG1 and CLN8 (TLC) lipid-sensing domain containing protein, TMO6 target of monopteros 6, DOF6 DNA binding with one finger 6, HSP70-2 heat shock protein 70-2, GSTF4 glutathione S-transferase F4, LRD3 lateral root development 3, ATGUS1 glucuronidase 1, NEN1 NAC45/86-dependent exonuclease-domain protein 1, ACI1 ALC-interacting protein 1, VCC vasculature complexity and connectivity, TOL4 TOM1-like 4, DA2 DA (large in Chinese) 2, MTPB1 metal tolerance protein B1.

Supplementary Figure 3 Construction and detection of the expression vectors. **(A)** Diagram of 35S::*BpPHD4* and 35S::*anti-BpPHD4* vectors. **(B)** Detection of the 35S::*BpPHD4* expression vector. **(C)** Detection of the 35S::*anti-BpPHD4* expression vector. M denotes the DL2000 marker, number 1 the positive plasmid containing *BpPHD4*, number 2 the negative control of double-distilled water, number 3 the negative control of empty plasmid, number 4 to 6 the targeted sequences of *BpPHD4*.

Supplementary Figure 4 Regeneration and identification of *BpPHD4* transgenic birch. **(A)** Mature birch zygotic embryos are infected with *Agrobacterium* EHA105. **(B)** Transgenic callus has formed on one of the cut sites in a zygotic embryo. **(C, D)** A transgenic cluster of shoots has formed from a callus and cultured in regeneration medium for 30 d. **(E, F)** Transgenic shoots are transferred to rooting medium and soil. **(G)** Agarose gel electrophoresis of DNA amplification obtained by PCR from NT and *BpPHD4* overexpressed transgenic birch. **(H)** Agarose gel electrophoresis of DNA amplification obtained by PCR from NT and *BpPHD4* repressed transgenic birch. M denotes the DL2000 marker, number 1 the plasmid control containing *BpPHD4*, number 2 the negative control of double-distilled water, number 3 the negative control of NT birch, number 4 to 6 the amplification of *HygR* in *BpPHD4* overexpression transgenic birch, number 9 to 11 the amplification of *HygR* in *BpPHD4* repression transgenic birch.

Supplementary Figure 5 Scanning electron microscopic observation on the primary vein of NT and *BpPHD4* transgenic plants.

Supplementary Figure 6 The relationships of modules and traits in *BpPHD4* overexpressed transgenic lines. PVD represents primary vein diameter, UE upper epidermis, LE lower epidermis, PT palisade tissue, ST spongy tissue, LT leaf thickness, NEC the number of epidermal cells, DSV the distance between secondary veins, NSV number of secondary veins, PVL primary vein length, IAPVSV angle between primary veins and secondary veins, LL leaf length, LA leaf area, LW leaf width.

Supplementary Table 1 Primers used for vector construction.

Supplementary Table 2 Primers used for validating *BpPHD4* transgenic birch.

Supplementary Table 3 The predicted information of putative HD-like TFs using InterProScan software.

Supplementary Table 4 Sequence alignment of HD-like genes in *Arabidopsis* and birch.

Supplementary Table 5 Family information of HD-like genes in birch.

Supplementary Table 6 Anatomical observation on the leaves of *BpPHD4* overexpressed and repressed transgenic lines.

Supplementary Table 7 Transcriptome data evaluation of *BpPHD4* overexpression transgenic lines.

Supplementary Table 8 Candidate genes regulated by *BpPHD4* identified from *BpPHD4* overexpression transgenic lines.
