# Supplementary material for: Identification of a potential homeodomain-like gene governing leaf size and venation architecture in birch
Source: Front Plant Sci. 2025 Jan 8;15:1502569. doi: 10.3389/fpls.2024.1502569 (PMC11751010; doi:10.3389/fpls.2024.1502569)
Supplement: Supplementary Figure 1 — Phylogenetic tree of HD-like superfamily genes in birch and Arabidopsis. Genes in KNOX family are used as an outgroup. The reliability of this phylogenetic tree is evaluated by the bootstrap value of 1000 replicates. Black solid triangle denotes the HD-like gene in Arabidopsis. Black solid circle denotes the HD-like gene in birch. [file DataSheet2.docx]

**Fig. S1 Phylogenetic tree of HD-like superfamily genes in birch and *Arabidopsis*.** Genes in KNOX family are used as an outgroup. The reliability of this phylogenetic tree is evaluated by the bootstrap value of 1000 replicates. Black solid triangle denotes the HD-like gene in *Arabidopsis*. Black solid circle denotes the HD-like gene in birch.

**Fig. S2** Co-expression network of HD-like genes and marker genes related to leaf vascular tissue. Pink rectangle represents the HD-like genes, orange circle the marker genes related to leaf vascular tissues, ATRLCK VI_A3 *Arabidopsis* receptor-like cytoplasmic kinase ATRLCK VI_A3, CML30 calmodulin-like 30, APL altered phloem development, PXL2 phloem intercalated with xylem-like 2, CDC2C CDC2CAT, APX5 ascorbate peroxidase 5, GATA20 GATA transcription factor 20, SMXL5 SMAX1-like 5, ATPP2-A9 phloem protein 2-A9, AT4G10360 TRAM, LAG1 and CLN8 (TLC) lipid-sensing domain containing protein, TMO6 target of monopteros 6, DOF6 DNA binding with one finger 6, HSP70-2 heat shock protein 70-2, GSTF4 glutathione S-transferase F4, LRD3 lateral root development 3, ATGUS1 glucuronidase 1, NEN1 NAC45/86-dependent exonuclease-domain protein 1, ACI1 ALC-interacting protein 1, VCC vasculature complexity and connectivity, TOL4 TOM1-like 4, DA2 DA (large in Chinese) 2, MTPB1 metal tolerance protein B1.

**Fig. S3 Construction and detection of the expression vectors.** a, Diagram of 35S::*BpPHD4* and 35S::*anti-BpPHD4* vectors. b, Detection of the 35S::*BpPHD4* expression vector. c, Detection of the 35S::*anti-BpPHD4* expression vector. M denotes the DL2000 marker, number 1 the positive plasmid containing *BpPHD4*, number 2 the negative control of double-distilled water, number 3 the negative control of empty plasmid, number 4 to 6 the targeted sequences of *BpPHD4*.

**Fig. S4 Regeneration and identification of *BpPHD4* transgenic birch.** a, Mature birch zygotic embryos are infected with *Agrobacterium* EHA105. b, Transgenic callus has formed on one of the cut sites in a zygotic embryo. c and d, A transgenic cluster of shoots has formed from a callus and cultured in regeneration medium for 30 d. e and f, Transgenic shoots are transferred to rooting medium and soil. g, Agarose gel electrophoresis of DNA amplification obtained by PCR from NT and *BpPHD4* overexpressed transgenic birch. h, Agarose gel electrophoresis of DNA amplification obtained by PCR from NT and *BpPHD4* repression transgenic birch. M denotes the DL2000 marker, number 1 the plasmid control containing *BpPHD4*, number 2 the negative control of double-distilled water, number 3 the negative control of NT birch, number 4 to 6 the amplification of *HygR* in *BpPHD4* overexpression transgenic birch, number 9 to 11 the amplification of *HygR* in *BpPHD4* repression transgenic birch.

**Fig. S5** Scanning electron microscopic observation on the primary vein of NT and *BpPHD4* transgenic plants.


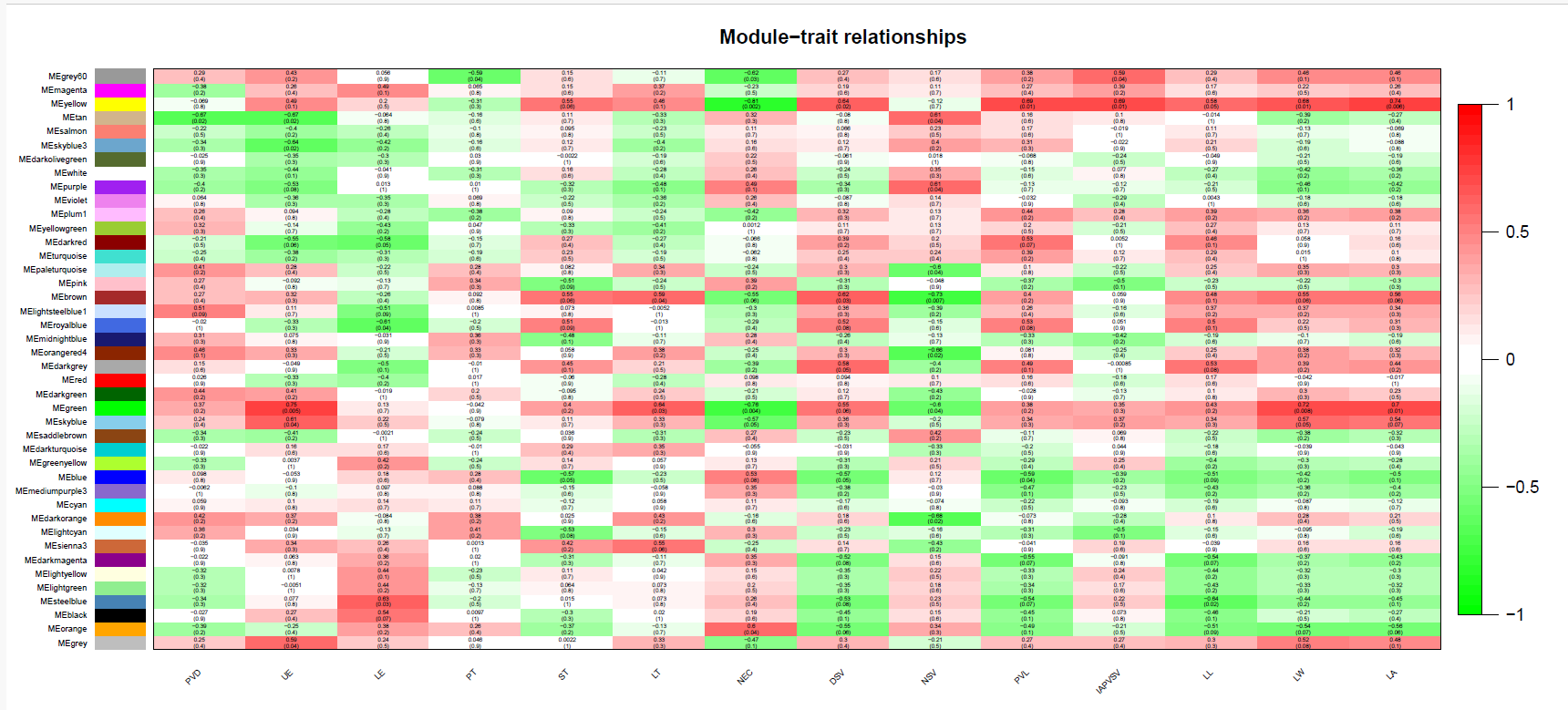


**Fig. S6 The relationships between modules and traits in *BpPHD4* overexpressed transgenic lines.** PVD represents primary vein diameter, UE rupper epidermis, LE lower epidermis, PT palisade tissue, ST spongy tissue, LT leaf thickness, NEC the number of epidermal cells, DSV the distance between secondary veins, NSV number of secondary veins, PVL primary vein length, IAPVSV angle between primary veins and secondary veins, LL leaf length, LA leaf area, LW leaf width.
